# Work-family interfaces and leaders’ knowledge hiding: underlying mechanisms and contingencies

**DOI:** 10.3389/fpsyg.2025.1666321

**Published:** 2025-11-06

**Authors:** Min Min, Kai Fang, Zhen Zhang, Ziyue Xu

**Affiliations:** 1 School of Supply Chain Management, Ningbo Polytechnic University, Ningbo, China; 2 Zhongkai University of Agriculture and Engineering, Guangzhou, China; 3 Business School, Ningbo University, Ningbo, China

**Keywords:** work-family conflicts, affective organizational commitment, self-esteem, knowledge hiding, innovative team leader, conservation of resources theory

## Abstract

**Objectives:**

The current study aims to examine the effects of work interference with family (WIF) and family interference with work (FIW) on leaders’ knowledge hiding, as well as the mediating role of affective organizational commitment and the contingent role of organization-based self-esteem.

**Methods:**

Data were collected through a three-wave survey from 137 new product team leaders in China, with a 2-week interval between waves to reduce common method bias.

**Results:**

Our findings indicate that FIW was positively related to knowledge hiding. This positive linkage was partially mediated by affective organizational commitment. Organization-based self-esteem weakens the impact of FIW on affective organizational commitment. In addition, as affective organizational commitment increased, the positive indirect effect of FIW on knowledge hiding becomes weaker. By contrast, the relevant results related to the effect of WIF were not significant.

**Conclusion:**

Extant research on micro-innovation has mainly highlighted the within-domain stressful effects of job conflicts but largely neglected their cross-domain mechanisms in shaping leaders’ knowledge behaviors. This study is one of the first to investigate how and when work-family interfaces influence top-down knowledge-hiding behavior. Practically, the findings provide guidance for organizations to design family-supportive and esteem-enhancing HR practices to reduce leaders’ knowledge hiding driven by FIW.

## 1 Introduction

Knowledge hiding - defined as individuals’ intentional attempts to conceal or withhold knowledge requested by others (Connelly et al., 2012) - precludes knowledge seekers from acquiring new insights and has emerged as a serious threat to organizational development (Arain et al., 2020; Peng et al., 2021; Aleksić et al., 2022). For instance, knowledge hiding has been linked to considerable productivity losses in organizations, with recent reports suggesting that such behavior may cost American companies up to $47 million in lost productivity (Nguyen et al., 2022). Moreover, numerous scholars contended that if organizations leave knowledge hiding problems unsolved, they are likely to experience lower levels of individual creativity (Arain et al., 2019; Chatterjee et al., 2021; Singh, 2019) and reduced team innovation (Fong et al., 2018; Zhang et al., 2022). To avoid potential innovation-related losses and risks, it is necessary to understand predictors leading to individuals’ knowledge hiding within organizations.

Individuals working in innovative organizations are required to simultaneously cope with organizational norms, client requirements, multiple role expectations, and parallel tasks (Zhao and Jiang, 2022). Given ever-increasing job demands faced by organizational members, work stressors arising from heavy job workloads have been highlighted as a critical predictor of knowledge hiding. Zhao and Jiang (2022) found that role stress (stemming from job role expectations) discourages employees to help others and positively contributes to their engagement in knowledge hiding. Additionally, drawing on the interpersonal nature of social job demands, scholars have demonstrated that various interpersonal stressors—such as task conflict, relationship conflict, and perceived injustice—can trigger employees’ knowledge hiding (Cao, 2022; Losada-Otálora et al., 2020; Peng et al., 2021; Semerci, 2019). Although prior studies have greatly improved our understanding of stressful effects of specific job demands on employees’ knowledge hiding, several issues in this line of research remain under-explored.

First, the above studies primarily highlighted within-domain (i.e., work domain) stressful effects of overloaded job demands on knowledge hiding. However, research on the work-family interface has argued that job demands can also spill over into family domain (Hall et al., 2010; He et al., 2024), leading to work interference with family that further backfires on employees’ work-domain behaviors (Frone et al., 1992; Xia et al., 2018). Moreover, extant studies have considered job demands as the sole source of work-domain stressors that stimulate employees’ knowledge hiding while overlooking the importance of demands originating from other domains (e.g., family demands). Kim et al. (2015) emphasized that employees’ growing family demands may be contradictory with their work roles and further interact with job demands to determine employees’ knowledge transfer intentions. Given significant demographic changes and fierce competition in innovative organizations, employees increasingly suffer from stressors of work-family imbalance (Byron, 2005, Kim et al., 2015). A cross-domain approach not only provides a more comprehensive understanding of stressful effects of job demands on knowledge hiding but also better match the life status of contemporary knowledge workers.

Second, recent reviews have highlighted that knowledge hiding has emerged as a fast-growing research domain with diverse theoretical perspectives, however, notable gaps remain in understanding leaders’ own knowledge-hiding behaviors (He et al., 2021). Leaders are widely recognized as playing the decisive role in fostering teamwork and to serve as role models who guide subordinates’ behaviors (Zhang and Min, 2024a). Top-down and leader-signaled knowledge hiding has been found to cause subordinates’ knowledge hiding, work withdrawal behavior, and work disengagement and it usually has severe implications for organizational outcomes (Arain et al., 2020; Offergelt et al., 2019; Xu et al., 2022) and harmful the performance of teams they manage (Nauman et al., 2025; Zhang and Min, 2024b). To further advance this important line of inquiry, the current study investigates how and when cross-domain conflicts influence leaders’ knowledge hiding.

Conservation of resources (COR) theory suggests that individuals strive to seek, maintain, and protect their valued resources. The loss of these resources can cause individuals to experience stress (Hobfoll, 1989). According to Xia et al. (2018), leaders who are juggling cross-domain conflicts may face a process of work resource drain (e.g., lacking energy to complete tasks). This process of resource loss undermines confidence in career prospects, thereby reducing leaders’ psychological bond with their organizations (i.e., affective commitment) (Lapointe et al., 2013). Therefore, leaders with low affective commitment may not voluntarily respond to subordinates’ requests and may engage in knowledge hiding to avoid further resource loss (Feng and Wang, 2019). To address how work–family conflicts influence leaders’ knowledge hiding, we explore the mediating role of affective commitment.

As COR theory suggests, higher levels of individual characteristic resources—especially self-esteem—help individuals resist stress. These resources enable them to cope with the negative psychological states caused by resource loss (Hobfoll, 1989). The perceived loss of resources from work–family conflicts may lead to different psychological and behavioral outcomes depending on one’s organization-based self-esteem. Organization-based self-esteem refers to the degree to which individuals believe themselves to be competent, important, and worthy organizational members (Pierce et al., 1989). Drawing on COR theory, we argue that organization-based self-esteem serves as a boundary factor that mitigates the stressful effects of work–family conflicts.

In summary, the current study makes two major contributions to extant organizational behavior research, particularly regarding the antecedents of leaders’ knowledge hiding. First, previous studies on knowledge hiding have mainly focused on the within-domain effects of job demands on peer knowledge hiding. This study is among the first to extend this view by identifying the cross-domain stressful mechanisms of job demands that predict leaders’ knowledge hiding. Second, drawing on the COR theory, we further offer a comprehensive explanation of how and when work-family conflicts influence leaders’ knowledge hiding by examining the mediating role of affective commitment and the moderating role of organization-based self-esteem.

## 2 Literature review and hypothesis development

### 2.1 Work-family conflict and knowledge hiding

According to research on work and family domains, work-family conflict is defined as the extent to which requirements related to work (or family) roles are incompatible with requirements related to family (or work) roles (Greenhaus and Beutell, 1985). Based on the sources resulting to inter-role conflict, work-family conflict can be classified into three forms: time-based conflict (i.e., excessive time devoted to one role limits an individual’s ability to fulfill the requirements of another role) (Netemeyer et al., 1996), strain-based conflict (i.e., strain derived from one role makes it difficult to satisfy the demands of another role) (Bruck and Allen, 2003), and behavior-based conflict (i.e., actions effective in one role are incompatible with those expected in another role) (Greenhaus and Beutell, 1985). As suggested by Frone et al. (1992), the concept of work family conflict exhibits a bidirectional property that includes work interference with family (WIF) and family interference with work (FIW).

The COR theory proposes that individuals under inter-role stresses always strive to maintain their resources (e.g., time, energy, and life status) and avoid resource loss (Hobfoll, 1989). Specifically, individuals who experience conflicts between work and family roles may encounter actual or threatened losses of work- and family-related resources, which trigger stress responses. Individuals tend to engage in behaviors that not only balance the demands of different domains (i.e., resource replacement) but also offset future resource loss (Grandey and Cropanzano, 1999; Kim et al., 2015).

When leaders experience high levels of WIF or FIW, they may face the threat of losing essential resources such as divorce and poor job performance (Xia et al., 2018). The resource loss in a process of juggling work and family roles makes leaders generate stress in both the work and family domains (Grandey and Cropanzano, 1999). Based on the propositions of COR, individuals with stresses will take actions that avert continuous loss of resource (Hobfoll, 1989). Knowledge hiding can serve as a means of maintaining resources. For example, knowledge hiding helps knowledge owners to avoid embarrassment of reduction of work resource (e.g., forfeiting knowledge power). As levels of WIF and FIW increase, leaders are more likely to hide knowledge for resource protection. Therefore, we propose the following hypothesis.

Hypothesis 1: WIF (a) and FIW (b) are positively associated with leaders’ knowledge hiding behavior.

### 2.2 Affective organizational commitment as a mediator

Meyer et al. (1990) conceptualized organizational commitment as a three-dimensional construct consisting of affective commitment (individuals’ emotional attachment to their working organization), continuance commitment (individuals’ awareness of the cost associated with leaving current organizations), and normative commitment (individuals’ sense of obligation to remain with their organization). In contrast to continuance and normative commitment, affective commitment is more responsive to work experience variables (e.g., organizational support) and exerts stronger effects on work-related outcomes (e.g., organizational citizenship behavior, performance, and turnover intentions). Building on these findings, the current study explores the mediating role of affective commitment in the relationship between work family conflict and knowledge hiding.

Work interference with family refers to the interference of work on family duties and creates stress associated with the the loss of family resources (e.g., divorce) (Xia et al., 2018). According to arguments of COR theory, to cope with stress derived from family resource loss, individuals may also engage in resource replacement behaviors (Hobfoll, 1989). That is, leaders may employ job resources in the work domain to withdraw the net loss of the family resources. The sacrificed work-related resources may aggravate the leaders’ inability to adequately perform the work role and lead them to perceive higher levels of work-related stress.

With regard to the case of FIW, leaders experiencing high levels of FIW find it difficult to meet their work demands. In this situation, they may be confronted with actual or potential net losses of work resources (e.g., nonproductive performance and job termination), which in turn induces leaders’ work-related psychological stress (Frone et al., 1992). Consequently, both WIF and FIW will give rise to leaders’ work-related psychological stress (Frone et al., 1992; Grandey and Cropanzano, 1999).

According to Zhang et al. (2012), work-related psychological stress can weaken individuals’ capacity to completely perform their work roles. Consequently, leaders’ inability to fulfill their work roles reduces their job-related rewards, such as promotions and bonuses (Aryee et al., 1999), which further diminishes leaders’ organizational attachment (Zhang et al., 2012). Therefore, both WIF and FIW weaken leaders’ affective commitment.

Hypothesis 2: WIF (a) and FIW (b) are negatively associated with leaders’ affective commitment.

Moreover, according to Meyer et al. (1990), leaders with high levels of affective commitment will deem organizations’ problems as their own. Knowledge-based psychological ownership, the state in which individuals feel as though the ownership of the knowledge is theirs, is a predictor of individual’s knowledge hiding behavior (Peng, 2013). When an individual put collective problems on the most important position, the individual is likely to pay more attention on others’ knowledge requests for problem solving rather than the self-interest. Knowledge-based psychological ownership decreases, leading to a lower level of knowledge hiding. Taken together, affective commitment is supposed to have a negative effect on knowledge hiding.

As noted previously, work family conflict (i.e., WIF and FIW) is hypothesized to impose negative influences on affective organizational commitment. That is, as the degree of work family conflict becomes higher, the level of affective organizational commitment will be lower. In addition, the decreased affective organizational commitment further enhances knowledge hiding. Therefore, it can be inferred that affective organizational commitment causes the indirect effects of work family conflict on knowledge hiding. Following this logic, we propose the following hypothesis:

Hypothesis 3: Affective organizational commitment is negatively associated with knowledge hiding.

Hypothesis 4: Affective organizational commitment mediates the relationships between (a) WIF and (b) FIW and knowledge hiding.

### 2.3 Organization-based self-esteem as a contingency

Organization-based self-esteem refers to the degree to which an individual believes him/herself to be competent, important and worthy as a member of the organization (Pierce et al., 1993). Pierce and Gardner (2004) suggested that individuals with higher levels of organization-based self-esteem usually have better work performance and possess a perception of meeting their demands via their roles in employing organizations. Following Ekrot et al. (2016), we further operationalize organization-based self-esteem as the extent to which a leader perceives him/herself as capable and significant within the organization.

As suggested previously, WIF and FIW can lead to leaders’ loss of work resource and in turn aggravate their work-related psychological stress. As a result, the increased stress undermines leaders’ affective organizational commitment. However, if a leader has a strong sense of organization-based self-esteem, the leader will be more capable of minimizing their work resource losses and experiencing lower levels of work stress. For instance, individuals with higher levels of organization-based self-esteem tend to exhibit greater composure and confidence, on which they can depend in dealing with problematic circumstances in work domains. Leaders are less disturbed by potential losses of work-related resources because of their confidence in coping with such a loss. Organization-based self-esteem can mitigate leaders’ work stress results from work resource loss. When organization-based self-esteem is present, WIF and FIW are less likely to reduce leaders’ organizational affective commitment by enhancing their work-related psychological stress. Accordingly, we propose the following hypothesis:

Hypothesis 5: Organization-based self-esteem moderates (weakens) the negative relationships between (a) WIF and (b) FIW and knowledge hiding.

Moreover, we have proposed that WIF and FIW exert an indirect positive effects on knowledge hiding through affective organizational commitment. Organization-based self-esteem moderates (weakens) the negative effect of WIF and FIW on affective organizational commitment. That is, the moderation of organization-based self-esteem leads to an additional (increased) strength of affective organizational commitment, which further reduces knowledge hiding. It is likely that organization-based self-esteem results in a conditional indirect effect of WIF and FIW on knowledge hiding through affective organizational commitment. Thus, we further propose the following hypothesis:

H6. The indirect positive effect of (a) WIF and (b) FIW on knowledge hiding via affective organizational commitment is moderated (weakened) by organization-based self-esteem.

## 3 Materials and methods

### 3.1 Sample and data collection

Innovative project leaders working in high-tech firms were identified as the target respondents of our survey. A sample was drawn from students who had enrolled in an advanced training program for innovation leadership at a national key university in China. Prior to data collection, all potential participants were informed that the study was conducted for research purposes and were assured of confidentiality and anonymity. A total of 253 respondents who were involved in new product innovation agreed to participate in our research.

As suggested by Peng (2013), self-report measure is an effective approach for evaluating individuals’ knowledge hiding behavior. However, self-report approach may also be subject to several biases (e.g., implicit theories and illusory correlations, leniency bias, and social desirability), which can contribute to common method bias (CMB) (Podsakoff et al., 2003). To reduce the noise caused by CMB, we followed recommendations of Zhu et al. (2019) and introduced a 2-week interval between the measurement of the predictor, mediator and outcome variables. In each wave, emails containing a unique number, a URL link to the web-based questionnaires, and a statement ensuring data confidentiality were sent to the target respondents. In the first wave (Time 1), project leaders were required to evaluate their level of work-family conflict, demographics, and information about their projects. In the second wave (Time 2), respondents were required to assess their affective organizational commitment and organization-based self-esteem. In the third wave (Time 3), leaders were asked to evaluate the frequency of their knowledge hiding behaviors. To improve the response rate, we sent reminder emails one week later. Finally, a total of 137 matched questionnaires were retained for subsequent analysis (response rate = 54.2%). Details of the leaders and their projects are described in Table 1.

**TABLE 1:** Information of project managers and projects (*N* = 137).

Item	Frequency	Percent
Gender		
Male	98	71.53%
Female	39	28.47%
Age		
Less than 30 years	24	17.52%
31–35 years	53	38.69%
36–40 years	41	29.93%
41 years or more	19	13.87%
Organizational tenure		
Less than 3 years	20	14.60%
4–6 years	33	24.09%
7–9 years	47	34.31%
10 years or more	37	27.01%
Product duration		
Less than 12 months	45	32.85%
13–18 months	24	17.52%
19–24 months	37	27.01%
25 months or more	31	22.63%
Team size		
Less than 10 members	44	32.12%
11–15 members	30	21.90%
16–20 members	34	24.82%
20 members or more	29	21.17%
Product type		
Information and communications	42	30.66%
New energy resources	34	24.82%
Chemical engineering	29	21.17%
Aerospace technology	17	12.41%
Others	15	10.95%

Percentages may not total exactly 100% due to rounding.

### 3.2 Measures

Work-family conflict. Based on Netemeyer et al. (1996), a 10-item scale was used to assess the dual direction values of leaders’ work family conflict. Five items of this measurement were related to work interfere with family, and the other five items were used to assess the degree of family interfere with work. Andres et al. (2012) employed this scale to assess leaders’ work family conflict, reporting a good reliability among items. Cronbach’s αs for WIF and FIW were 0.936 and 0.945, respectively.

#### 3.2.1 Leaders’ knowledge hiding

We originally adopted the 12-item scale developed by Connelly et al. (2012) to measure the three forms of knowledge hiding behavior (i.e., evasive hiding, playing dumb, and rationalized hiding). However, in line with recent empirical evidence suggesting that a shortened four-item version provides comparable validity (Wang et al., 2019; Jiang et al., 2022), we used four items in the present study. To better match the project settings, the items were started with a situation: “For a moment when one of your subordinates requested knowledge from you and you refused.” Cronbach’s α for knowledge hiding was 0.922.

#### 3.2.2 Affective organizational commitment

We used a five-item scale to evaluate affective organizational commitment. The five items were taken from the eight original items of Meyer et al. (1990) and two of them were reversely worded. Project leaders were required to evaluate the extent to which they were emotionally attached to their employing organizations and willing to solve organizations’ problems. Cronbach’s α for affective organizational commitment was 0.880.

#### 3.2.3 Organization-based self-esteem

A four-item scale developed by Ekrot et al. (2016) was adopted to assess leaders’ organization-based self-esteem. Project leaders were asked to evaluate the extent to which they believe themselves to be valuable and important as a project leader. Cronbach’s α for organization-based self-esteem was 0.880.

In the above scales, each item was rated on a seven-point Likert-type scale ranging from 1 (strongly disagree) to 7 (strongly agree).

#### 3.2.4 Control variables

According to previous studies, individuals’ demographic characteristics of individuals (e.g., age, gender, and tenure) may have influences on their knowledge hiding behaviors (Huo et al., 2016; Peng, 2013). Additionally, project managers’ behaviors may vary depending on their work pressure (Xia et al., 2018). Turner and Mariani (2016) argued that, in contrast to small-size projects, managers working in large-size projects may experience greater pressure. It is necessary to take into account project features when study behaviors of leaders. Therefore, age (continuous variables in years), gender (coded as dummy variable; 1 = male, 0 = female), tenure (continuous variables), project team size (continuous variables in years), and product duration (continuous variables in months) were considered to be control variables of this study.

### 3.3 Common method bias

As mentioned previously, single source self-report data may lead to CMV. Time-lagged design for data collection is an effective ex-ante remedy for avoiding CMV. In addition, we also used Harman’s one-factor test as an ex post remedy for CMB (Podsakoff and Organ, 1986). The results of one factor analysis showed that eigenvalues of five factors exceeded 1.0, which is equal to the number of constructs. The contribution values of five factors to the variance were 25.156% (lower than the criterion of 50%), 24.165%, 12.470%, 10.763%, 8.731%, respectively. This result implied that no single factor could account for the majority of the covariance. Therefore, common method bias was not a big concern to our research.

## 4 Results

This study employed PLS-SEM to examine our theoretical model for two concerns. SmartPLS 4.1 software was used for examining the hypotheses depicted in our research model. We adopted the outer model to estimate the reliability and validity of each construct and applied the inner model to examine the assumptions for direct and moderating effects (Hair et al., 2011).

### 4.1 Measurement model

To examine the measurement model, we calculated the reliability, convergent, and discriminant validity of the survey data. Table 2 showed the Cronbach’s coefficient alphas, composite reliability (CR), average variance extracted (AVE), and factor loadings for items. According to Benitez et al. (2020), the minimum value of factor loadings should exceed the recommended threshold of 0.707. One item from the affective organizational commitment scale was removed due to a low factor loading (0.52). All the Cronbach’s coefficient alphas and CR values of studied variables are greater than the recommended threshold of 0.7 (Bagozzi and Yi, 1988). In addition, the minimum value of AVE was 0.647, surpassing the recommended threshold of 0.5. Thus, the results indicated good reliability and convergent validity of the data. Moreover, Fornell-Larker criterion was employed to assess the discriminant validation. According to Table 3, square roots of AVE for latent constructs were obviously greater than their relevant coefficients of cross-correlations, verifying good discriminant validity of the measurement model.

**TABLE 2:** The indices for construct reliability and convergent validity.

Construct/item	Factor loadings
Work interference with family (WIF)	
Cronbach’s α = 0.936, CR = 0.936, AVE = 0.746	
WIF1: the demands of my work interfere with my home and family life.	0.807
WIF2: the amount of time my job takes up makes it difficult to fulfill family responsibilities.	0.917
WIF3: things I want to do at home do not get done because of the demands my job puts on me.	0.881
WIF4: my job produces strain that makes it difficult to fulfill family duties.	0.860
WIF5: due to work-related duties, I have to make changes to my plans for family activities.	0.850
Family interference with work (FIW)	
Cronbach’s α = 0.945, CR = 0.944, AVE = 0.778	
FIW1: the demands of my family or spouse/partner interfere with work-related activities.	0.886
FIW2: i have to put off doing things at work because of demands on my time at home.	0.843
FIW3: things I want to do at work don’t get done because of the demands of my family or spouse/partner.	0.958
FIW4: my home life interferes with my responsibilities at work such as getting to work on time, accomplishing daily tasks, and working overtime.	0.910
FIW5: family-related strain interferes with my ability to perform job-related duties.	0.805
Organization-based self-esteem (OBSE)	
Cronbach’s α = 0.947, CR = 0.949, AVE = 0.822	
OBSE1: as a project manager I am important in this company.	0.986
OBSE2: as a project manager I am taken seriously in this company.	0.804
OBSE3: i am trusted as a project manager in this company.	0.939
OBSE4: i am valuable as a project manager in this company.	0.888
Affective organizational commitment (AOC)	
Cronbach’s α = 0.880, CR = 0.881, AVE = 0.647	
AOC1: this organization has a great deal of personal meaning to me.*	0.520
AOC2: i would be very happy to spend the rest of my career with this organization.	0.881
AOC3: i really feel as if this organization’s problems are my own.	0.727
AOC4: i do not feel a strong sense of belonging to this organization.	0.801
AOC5: i do not feel emotionally attached to this organization.	0.803
Project managers’ knowledge hiding (PMKH)	
Cronbach’s α = 0.922, CR = 0.917, AVE = 0.755	
For a moment when one of your subordinates requested knowledge from you and you refused:	
PMKH1: i agreed to help him/her but never really intended to.	0.943
PMKH2: i offered him/her some other information instead of what he/she really wanted	0.942
PMKH3: i said that I was not very knowledgeable about the topic.	0.847
PMKH4: i said that I would not answer his/her questions.	0.741

*One item from the affective organizational commitment scale was removed due to a low factor loading (0.52), which did not meet the recommended threshold of 0.707 (Benitez et al., 2020).

**TABLE 3:** The indices for discriminant validation.

Constructs	WIF	FIW	OBSE	AOC	PMKH
1. WIF	0.864	–	–	–	–
2. FIW	0.069	0.882	–	–	–
3. OBSE	0.510	−0.056	0.907	–	–
4. AOC	0.082	−0.222	0.089	0.805	–
5. PMKH	0.086	0.328	−0.044	−0.299	0.869

Diagonal elements show the square root of average variance extracted (AVE) for the corresponding construct. FIW, family interference with work; WIF, work interference with family; OBSE, organization-based self-esteem; AOC, affective organizational commitment; PMKH, project managers’ knowledge hiding.

### 4.2 Structural model

**FIGURE 1:**
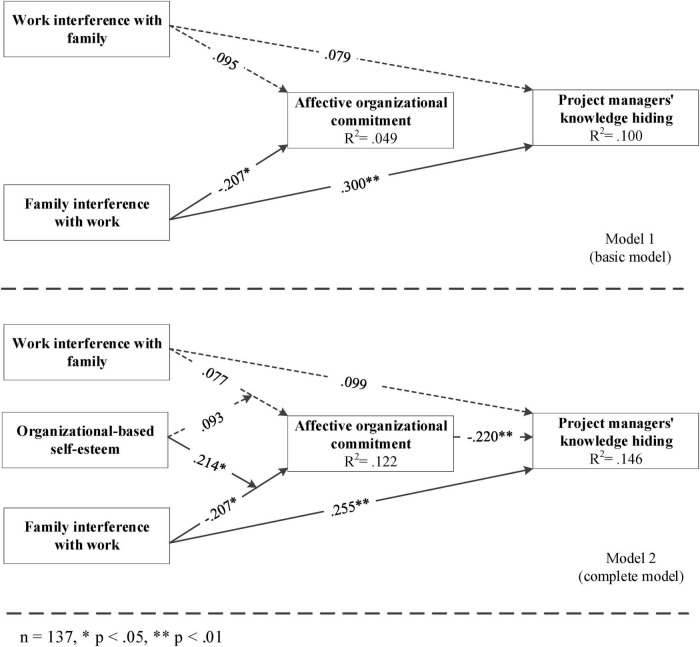
Models used for hypothesis testing.

We used a bootstrapping method with 5,000 steps to calculate the effect size for each relationship. According to Cohen’s (1988) criteria, an effect size above 0.1 is small, 0.3 is moderate, and 0.5 is strong, providing a practical interpretation of path magnitudes. Figure 1 shows the path coefficients of relationships between studied variables in two models. In Model 1 (baseline model), the influences of WIF on knowledge hiding and affective organizational commitment were null (β_H1a_ = 0.079, non-significant; β_H2a_ = 0.95, non-significant), rejecting H1a and H2a. By contrast, FIW had a moderate positive effect on knowledge hiding (β_H1b_ = 0.300, *p* < 0.01) and a small-to-moderate negative effect on affective commitment (β_H2b_ = −0.207, *p* < 0.05), supporting H1b and H2b.

As proposed previously, affective organizational commitment was negatively related to knowledge hiding and mediated the relationships between (a) WIF and (b) FIW and knowledge hiding. According to path coefficients displayed in Model 2 (the complete model), the association between affective organizational commitment and knowledge hiding was significantly negative (β_H3_ = −0.220, *p* < 0.05), supporting H3. This represents a small-to-moderate effect, suggesting that leaders’ affective organizational commitment plays a meaningful role in reducing knowledge hiding behavior. Moreover, the indirect effect of WIF and FIW on knowledge hiding through affective organizational commitment were −0.020 (β_H4a_, non-significant) and 0.045 (β_H4b_, *p* < 0.05), respectively. H4a was rejected and H4b was supported, indicating a partial mediation for FIW.

**FIGURE 2:**
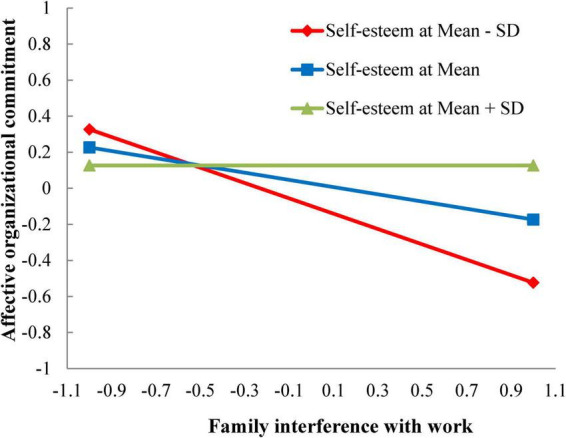
Interaction of FIW and organization-based self-esteem for knowledge hiding.

According to Model 2 (complete model), the effect of the product term between WIF and organization-based self-esteem was null (β_H5a_ = 0.093, non-significant), rejecting H5a. Given that H5a (moderation) acts as the precondition of H6a (moderated mediation), H6a was also rejected. As expected, organization-based self-esteem significantly moderated the FIW-knowledge hiding relationship (β_H5b_ = 0.214, *p* < 0.05), showing a small-to-moderate buffering effect, thereby supporting H5b. Following the suggestions of Aiken et al. (1991), the simple slope analysis of the moderating effects of organization-based self-esteem was described in Figure 2. To test the hypothesized moderated mediation (H6b), the bootstrapping method recommended by Preacher et al. (2007) was conducted.

We also computed the conditional indirect effects of FIW on knowledge hiding via affective organizational commitment across different levels of organization-based self-esteem (i.e., Mean−1SD, Mean and Mean+1SD). It was found that the indirect effect size of FIW on knowledge hiding significantly varied according to different levels of organization-based self-esteem (Mean−1SD = 2.77, significant; Mean = 4.00, significant; Mean+1SD = 5.25, non-significant). Therefore, H6b was supported.

## 5 Discussion and conclusion

### 5.1 Theoretical contribution

To our knowledge, this study is the first to explore the conflict-related antecedents of leaders’ counterproductive behavior in the context of innovative project teams. Therefore, our findings can contribute to extant literature on organizational behavior in several ways. First, this study enriches the understanding of the antecedents of leaders’ knowledge hiding. Studies have explored the consequences of leaders’ knowledge hiding (Arain et al., 2019, 2020; Singh, 2019; Zhang and Min, 2024a), whereas quantitative evidence on its antecedents remains scarce. Our study indicates that family interference with work and affective organizational commitment are significantly associated with knowledge hiding. These conclusions can enrich the literature on managing leaders’ knowledge hiding in projects. Moreover, research on knowledge hiding has demonstrated that interpersonal and task conflicts sourcing from excessive job demands predict knowledge hiding (Losada-Otálora et al., 2020; Peng et al., 2021; Semerci, 2019; He et al., 2024). In addition to work domain conflicts, job demands also lead to work family interference that causes problems of knowledge hiding. Our findings verified the differential effects of bidirectional work family conflicts on leaders’ knowledge hiding, therefore providing empirical evidence of cross-domain effects of work demands.

Second, this study extends our knowledge of the role played by affective commitment in interpreting work family conflict-knowledge hiding relationship. Previous studies have made efforts to understand the mediating role of employees’ state of being toward work in the relation between various types of conflict and knowledge hiding, such as individuals’ positive wellbeing (Losada-Otálora et al., 2020), envy (Peng et al., 2021), and emotional exhaustion (Zhao and Jiang, 2022). However, the factors above mainly focus on the state being toward work. Our understanding about the role of the individuals’ state of being toward organizations remains unknown. The findings indicate that organizational commitment mediates the positive relation between FIW and knowledge hiding, offering complementary insights into the underlying mechanisms linking conflict and knowledge hiding.

Third, this study verifies the additional proposition of COR in the field of knowledge hiding by examining the moderating roles of organization-based self-esteem in the direct and indirect paths between work family conflict knowledge hiding. COR argued that self-esteem, a typical form of individual characteristic resource, is important for stress resistance (Hobfoll, 1989). According to our findings, organization-based self-esteem weakens the direct effect of FIW on affective organizational commitment. Additionally, as organization-based self-esteem increases, the indirect effect of FIW on knowledge hiding decreases. These results confirm and extend the applicability of COR theory in knowledge management research.

Fourth, while prior research on cross-domain conflicts has emphasized the negative influence of work–family conflicts on project professionals’ creative and helping behaviors (Lin et al., 2022; Novieto and Kportufe, 2022; Yoon et al., 2024), our results suggest that these detrimental effects may not necessarily extend to counterproductive behaviors. Specifically, we found that the direct effect of WIF on leaders’ knowledge hiding (H1a) was not significant. One possible explanation lies in cultural context. In Eastern societies such as China, work tends to be highly prioritized over family responsibilities due to cultural values and economic insecurity (Zhang et al., 2012). In such contexts, using knowledge hiding as a strategy to compensate for the loss of family resources by preserving work-related resources becomes less necessary. Moreover, because knowledge hiding can undermine interpersonal trust and relationships at work (Zhang and Min, 2024a), cultural norms emphasizing work centrality may further discourage leaders from engaging in such behaviors, even when they experience work–family interference. Consequently, our findings suggest that cultural values may function as boundary conditions that help buffer the translation of family–work stressors into counterproductive knowledge behaviors.

### 5.2 Empirical implications

Our findings can also provide managerial implication for managing leaders’ knowledge hiding in innovative project teams. First, compared to WIF, FIW is more likely to result in leaders’ knowledge hiding behavior. It is necessary for organizations engaged in innovation projects to adopt family-supportive policies (e.g., welfare regimes and flexible working time) to mitigate the family work conflicts originating from family roles. Additionally, more supportive practices can be adopted according to the situations of a specific project. For example, if a forthcoming project requires its project manager to experience a long-term separation, organizations are encouraged to extend the interval between the former project and latter project, which allows the project manager to have plenty of time to spend with family (Turner et al., 2008).

In terms of results, affective organizational commitment reduces knowledge hiding and mediates the indirect effect of FIW on knowledge hiding. When designing knowledge management practices to mitigate knowledge hiding, it is suggested to consider their effectiveness in fostering leaders’ affective commitment. This is particularly relevant for innovative project teams, where high task interdependence and rapid problem-solving demand a strong emotional attachment from managers to their organizations, which in turn fosters continuous knowledge exchange (Xia et al., 2018). For example, the effectiveness of knowledge management practices can benefit from enriching individuals’ perceived organizational support, which augments individuals’ affective commitment (Jeung et al., 2017).

Moreover, organization-based self-esteem serves as a contingency in the relationship between FIW and knowledge hiding. Leaders are more effective in dealing with negative emotions arising from family work conflict. When firms assign managers for projects, individuals who strongly believe themselves to be competent, important and valued in their employing organizations should be selected. Moreover, when training leaders’ expertise and management skills, organizations are encouraged to offer programs to enhance leaders’ organization-based self-esteem.

Finally, although our study was conducted within the Chinese context, these implications also extend to global organizations. Multinational firms face increasingly diverse workforces within which family–work conflicts are prevalent. To mitigate knowledge hiding in such organizations, it is necessary to adopt HR practices that acknowledge cultural diversity and address cross-border work–family challenges. Specifically, HR policies such as international relocation support, cultural adjustment training, and global mobility programs can reduce strain and help leaders remain effective across borders (Hsiao, 2022; Mendenhall and Reiche, 2025), thereby alleviating family–work conflicts, thereby alleviating family–work conflicts. Accordingly, global organizations are encouraged to implement these practices to foster a more collaborative and sustainable knowledge-sharing environment.

### 5.3 Limitations and future research

Similar to previous studies, our study also has several limitations. First, although self-report surveys are widely used in knowledge hiding research and have been recommended as an efficient method to capture different forms of knowledge hiding behavior (Peng, 2013; Connelly et al., 2012), they may still be subject to social desirability bias given the sensitivity of the construct. To reinforce the robustness and generalizability of our conclusions, future studies are strongly encouraged to complement self-reports with alternative approaches, such as behavioral experiments or observational methods.

Second, affective organizational commitment did not explain the total effect of WIF on knowledge hiding (partial mediation). To better fertilize our knowledge about the whole story of how work family conflict leads to knowledge hiding, scholars should explore other potential variables interpreting the residual effects of WIF from more theoretical lenses.

Third, our study focuses on project managers’ general knowledge hiding in response to subordinates’ requests and does not distinguish between explicit and tacit forms of hidden knowledge. While our findings indicate that general knowledge hiding can be reduced through affective organizational commitment, this approach cannot capture the potentially differential effects of predictors on distinct types of knowledge hiding. Future research could provide more fine-grained insights by explicitly separating explicit knowledge hiding from tacit knowledge hiding, thereby revealing whether their antecedents vary across different contexts.

Fourth, the data used for hypothesis testing were collected from project managers in China. Given the substantial cultural distance between China and Western countries, the conclusions may not be fully generalizable to project managers outside mainland China. In particular, the differing cultural values associated with collectivism and individualism may lead to distinct behavioral responses to family–work conflicts. Therefore, future research is encouraged to replicate our findings in Western contexts or to conduct cross-cultural comparative studies to enhance the generalizability of the results.

Finally, our sample data were collected from project leaders in China. Therefore, our conclusions and implications may not be applied to the situation of project leaders outside mainland China. Further studies should test the findings of our work in a different national and cultural background, especially in western countries.

### 5.4 Conclusion

This study examines how and when cross-domain conflicts influence leaders’ knowledge hiding by adopting conservation of resources theory as a theoretical perspective. The findings highlight that family interference with work, rather than work interference with family, increases leaders’ knowledge hiding through reduced affective organizational commitment, while organization-based self-esteem mitigates this indirect effect. These results advance theoretical understanding by revealing the cross-domain and cultural boundary conditions under which work–family stressors shape counterproductive knowledge behaviors. Practically, the study provides actionable insights for organizations to design family-supportive and esteem-enhancing HR practices that reduce leaders’ knowledge hiding and foster a more open and collaborative knowledge-sharing environment.

## Data Availability

The raw data supporting the conclusions of this article will be made available by the authors, without undue reservation.
